# A study protocol for a double-blinded, randomised, placebo-controlled trial on the use of encapsulated FMT for reducing the side effects of HSCT: the HSCT-BIOME study

**DOI:** 10.1186/s12885-025-14057-4

**Published:** 2025-04-10

**Authors:** Anna Li, Sam P. Costello, Robert V. Bryant, Sarah Haylock-Jacobs, Craig Haifer, Cindy Lee, David Yeung, Pratyush Giri, Danielle Blunt, Joanne B. Bowen, Feargal J. Ryan, Angelina Yong, Hannah R. Wardill

**Affiliations:** 1 School of Biomedicine, The University of Adelaide, Adelaide, Australia; 2 Supportive Oncology Research Group, Precision Cancer Medicine, The South Australian Health and Medical Research Institute, Adelaide, Australia; 3 BiomeBank, Adelaide, Australia; 4 Adelaide Medical School, The University of Adelaide, Adelaide, Australia; 5 Department of Gastroenterology, Basil Hetzel Institute, The Queen Elizabeth Hospital, Adelaide, Australia; 6 Department of Gastroenterology, St Vincent’s Hospital Sydney, Sydney, Australia; 7 School of Clinical Medicine, University of New South Wales, Sydney, Australia; 8 Department of Haematology, The Royal Adelaide Hospital, SA Health, Adelaide, Australia; 9 College of Medicine and Public Health, Flinders University, Adelaide, Australia; 10 Level 5S, SAHMRI, North Terrace, Adelaide, 5000 SA Australia

**Keywords:** Autologous haematopoeitic stem cell transplantation, Capsule fecal microbiota transplantation, Peri-HSCT fecal microbiota transplantation

## Abstract

**Background:**

The composition of the gut microbiota both prior to and after haematopoietic stem cell transplantation (HSCT) is increasingly implicated in the outcomes of HSCT, including infections, poor immune reconstitution and disease relapse. Faecal microbiota transplantation (FMT) offers a potential strategy of supporting the gut microbiota and improve HSCT outcomes. Although FMT has been investigated in HSCT recipients, it has largely been evaluated therapeutically for indications such as infection, or once immunocompetency is regained.

**Methods:**

Peri-HSCT FMT (i.e. before and after HSCT) will be administered to eligible participants (adults undergoing autologous HSCT for a haematological malignancy) over two courses, with the first delivered immediately prior to conditioning and the second starting when ANC > 0.8. Following an open-label, safety run in (N = 5), peri-HSCT FMT will be evaluated for its efficacy in 51 participants, randomised 2:1 to FMT or placebo. The primary outcome is the proportion of participants who develop severe gastrointestinal toxicity defined by 3 consecutive days of severe diarrhoea (Bristol Stool Chart 6+), at a frequency of 4 + bowel movements/day within 3 weeks of HSCT. Safety is defined as the incidence of treatment-emergent adverse events (TE-AEs). Tolerability is defined as the incidence of TE-AEs and adherence to FMT.

**Discussion:**

The HSCT-BIOME study is a multi-centre, double-blind, randomised placebo-controlled trial designed to determine the tolerability, safety and efficacy of orally-administered encapsulated FMT to promote the stability of the gastrointestinal microenvironment for HSCT recipients. Peri-HSCT delivered FMT is hypothesised to promote microbial composition both before and following HSCT. Thus, the study will determine if administration of FMT post-HSCT during the neutropenic phase will enhance efficacy.

**Trial registration:**

ACTRN12624001104549. Date of registration: September 19, 2024 (prospectively registered).

## Background

Haematopoietic stem cell transplantation (HSCT) is a common treatment for haematological malignancies. It involves the use of high dose chemotherapy +/- total body irradiation (TBI), both of which serve to ablate the patient’s bone marrow, which is then reconstituted by infusion of stem cells collected from the patient (autologous HSCT) or a matched donor (allogeneic HSCT). Both auto- and allo-HSCT recipients experience a range of transplant related toxicities randing from infections, diarrhoea, mucositis, malnutrition, pain, to graft versus host disease (GvHD, allo-HSCT only) [1–3]. These require various interventions and treatment, which often serve to relieve symptoms but do not address the core biological drivers of these complications.

Recently, the contribution of the ecosystem of micro-organisms residing in the gastrointestinal tract – the gut microbiota – to HSCT complications has been increasingly recognised. Indicators of microbial disruption both before and after HSCT have been identified to predict a range of complications. For example, patients with low microbial diversity and high abundance of pathogenic microbial taxa were more likely to develop blood stream infections (BSI) after HSCT. In fact, the pre-chemotherapy gut microbiota was able to predict infection risk with a sensitivity of 90% [4]. Similarly, detrimental microbial attributes that are consistently reported in HSCT recipients (e.g. low diversity and pathogen expansion) have been identified to increase the risk of BSI, pulmonary infections, perturbed immune reconstitution, malnutrition, graft-versus-host disease and relapse [1, 5, 6].

With current evidence implicating gut microbiota disruption in HSCT-associated complications, strategies designed to stabilise or restore the gut microbiota have emerged. Faecal microbiota transplantation (FMT) is a method by which healthy gut micro-organisms are collected, processed and administered to a recipient to restore or change their resident microbial community [7]. FMT has been trialled in HSCT recipients with *Clostridium difficille infection* (CDI), multidrug resistant bacteria (MDB) and severe, steroid-refractory GvHD with considerable success [8, 9]. A recent systematic review by Malard indicates that when used therapeutically, FMT induced complete response in ~ 40% of people with GvHD [9]. These benefits have been attributed to FMT’s ability to restore eubiosis and in turn, stimulate production of beneficial metabolites such as short chain fatty acids (SCFA), which maintain the intestinal barrier and modulate the immune system [10].

One of the major challenges in the routine use of FMT in HSCT recipients is its method of delivery. Conventionally, FMT is administered colonoscopically, via enema or nasogastric tube. These methods of administration are complex and often contraindicated in immunocompromised HSCT recipiens, especially those with friable colonic mucosa. With a recent landmark trial investigating the use of orally administered encapsulated FMT in HSCT recipients by Rashidi and colleageues [11], and demonstration of similar efficacy of encapsulated FMT to colonoscopically delivered formations in other clinical settings (e.g. CDI and Inflammatory Bowel Disease), this formulation should provide a more feasible strategy of delivering FMT to vulnerable patient cohorts [12]. Rashidi reported that encapsulated FMT delivered to immunocompetent patients was safe and ameliorated intestinal dysbiosis, however, it was not able to reduce the incidence of BSI in allo-HSCT recipients [11]. This result may reflect the timing of FMT delivery, which although earlier than previous studies, may not have appropriately supported the gastrointestinal microenvironment early enough in the aetiology of infection [13].

Administering FMT acutely after HSCT, when patients are more immunocompromised, has been largely avoided due to perceived risks of bacterial translocation across a damaged gut mucosa, and thus infection. However, FMT has consistently been shown to restore colonisation resistance and actually prevent pathogenic single strain domination events such as in the context of CDI. While blood stream infection (BSI) in HSCT patients are complicated by association with gut mucosal injury, it is similarly preceded by domination events by strains such as *Enterococcus faecium* or *Escherichia coli* [13]. Further, a recent report highlighted that offending strains in BSI events in immunocompromised patients with colonic GvHD were not detected in FMT donations [14]. Likewise, studies of FMT for HSCT have insofar not been reported to increase the incidence of BSI and has limited adverse events when strict donor criteria are followed [15]. Thus, with improved delivery methods, which overcome the challenges of traditional FMT for early use in HSCT [16], it is pertinent to act on the idea that FMT may be safe for use in neutropenic patients with or without friable bowel, and most importantly, may restore the much needed benefits of colonisation resistance provided by a diverse, functioning microbiome early after the depletion of an immune system.

Here, we present a protocol to investigate the safety and efficacy of orally-administered, encapsulated FMT in auto-HSCT recipients. The critical point of difference in this protocol compared to existing literature is the peri-HSCT delivery of FMT, with FMT delivered before conditioning therapy and after HSCT whilst the patient remains neutropenic (ANC ≥ 0.8). It is hypothesised that this approach will improve the efficacy of FMT by improving gut microbiota composition prior to HSCT, and support its stability early in the aetiology of transplant complications.

## Methods

### Study aims

- Determine if encapsulated FMT reduces the proportion of participants that develop severe diarrhoea within 3 weeks of HSCT.
- Determine adherence to encapsulated FMT.
- Determine rates of FMT treatment emergent adverse events.

### Study objectives and outcome measures

**Table:** 

Objectives	Outcome measures
Determine prophylactic efficacy of FMT on diarrhoea	Proportion of patients that develop severe diarrhoea determined by 3 consecutive days of BSC6 + at a frequency of 4 + bowel movements/day above baseline within 3 weeks of HSCT * Note: BSC and frequency will also be evaluated individually
Determine therapeutic efficacy of FMT on diarrhoea	Duration of severe diarrhoea defined as BSC6 + at a frequency of 4 + bowel movements/day above baseline
Determine clinical impact of FMT	Changes in body weight (kg) Incidence of fever (body temp > 37.8oC) Incidence of blood stream infections Use of supportive care interventions Duration of hospitalisation
Determine safety of FMT	Incidence of TE-AEs
Determine adherence to FMT administration	Number of capsules taken
Determine effect of FMT on mucosal barrier integrity	Plasma citrulline concentrations
Determine colonisation and function of donor FMT	Gut microbiota composition and diversity determined using bacterial genome sequencing on stool samples and SCFA analyses in plasma
Determine changes in symptom burden	ESAS-r-CS

### Trial design

To determine the safety and feasibility of peri-HSCT encapsulated FMT to improve outcomes of HSCT, the HSCT-BIOME Study will involve two stages:

Stage 1 – Safety run in: Open-label study in N: 5 participants to identify any TE-AEs and confirm adherence to the protocol. There will be a planned review by the Safety Monitoring Committee following Stage 1 to review outcomes and reported AEs before enrolment of participants in Stage 2 of the study.

Stage 2 – Efficacy trial: Double-blind, randomised, placebo-controlled trial to determine the clinical efficacy of the peri-HSCT encapsulated FMT.

### Patient and public involvement

Consumer representatives, identified through local advocacy group Cancer Voices SA, were consulted with respect to the design and implementation of this study. Specific design points dictated by consumers were the number of capsules deemed tolerable to take per day and methodology for data collection. Consumers provided input for all participant material, assisting with the phrasing of treatment side effects, FMT and other complexities of the trial to ensure it was accessible to a lay audience. Community organisations such as Leukaemia Foundation and Myeloma Australia will be involved to ensure these data are communicated with consumers.

### Study setting

- Royal Adelaide Hospital, Adelaide, Australia.
- St Vincent’s Hospital, Sydney, Australia.

Further sites may be added once the trial has commenced. These will remain restricted to Australia.

All samples will be processed locally, but stored at the South Australian Health and Medical Research Institute or at BiomeBank (Adelaide, South Australia).

### Participants and study recruitment

The HSCT-BIOME trial will recruit eligible participants scheduled for conditioning chemotherapy prior to autologous HSCT for a haematological malignancy (Table 1).

This is a pilot study aiming to recruit N: 51 participants. This will adequately power the study to detect a 40% absolute reduction (80–40%) in the proportion of participants that meet the primary outcome of the study (alpha: 0.05, beta: 0.2, power 80%).

Potentially eligible participants will be identified at multidisciplinary team meetings (MDTs), hospital admissions and outpatient clinics The study will be introduced to the participant by their clinician, and provided with the PICF and the study will be introduced to the participant by their clinician. Between stem cell mobilisation and providing consent for their HSCT, participants will be given the PICF and consent will be collected at the same time as consenting for HSCT.

Once consented to the study, the participant is assigned a Study ID which will be used to identify all study material.

**Table 1:** Inclusion and exclusion criteria

Inclusion	Exclusion
1. Age ≥ 18 years	1. Pre-existing gastrointestinal disease including Crohn’s disease, ulcerative colitis
2. Diagnosis of multiple myeloma, lymphoma or another haematological malignancy to be treated with auto-HSCT	2. Unable to swallow capsules
3. Scheduled to receive conditioning chemotherapy (+/- TBI) prior to auto- HSCT	3. Pregnancy
4. Able to provide written informed consent and follow all clinical trial related procedures (translator to be provided for people of culturally and linguistically diverse (CALD) backgrounds)	4. Nut allergy or anaphylactic food allergy
	5. Uncontrolled vomiting or oral mucositis that may impact swallowing (determined by participant) **
	6. Fever (body temp > 37.8oC) **

**Eligibility criteria to be reviewed before post-HSCT FMT intervention

### Investigational product

#### Intervention

Encapsulated, lyophilised FMT administered peri-HSCT (prior to and following HSCT). Pre-HSCT FMT will be administered 1 week prior to conditioning chemotherapy. Post-HSCT FMT will be delivered when ANC reaches 0.8 or higher. Each course is administered as 36 capsules taken at any time of the day (one “course” contains 25 g of stool) (Table 2).

**Table 2:** Timing and delivery of peri-HSCT encapsulated FMT

Course	Starting indication	Number of capsules per day	Duration
1 (pre-HSCT)	1 week prior to start of conditioning chemotherapy	6 (3 BD)	6 days
2 (post-HSCT)	ANC>/= 0.8	6 (3 BD) for 2 days followed by 2 capsules (once daily)	14 days

#### Preparation of FMT

Donor stool will be sourced from healthy volunteers and rigorously screened for infections, antibiotic resistant bacteria, and all other criteria specified by the Australian Therapeutic Goods Administration (TGA) [17]. Standard operating procedures for the collection, preparation, storage and release of FMT are established at BiomeBank. All processes regarding BiomeBank FMT are conducted within a comprehensive quality control system in line with TGO 105 and principals of GMP.

#### Comparator

Placebo capsules (Stage 2 only).

### Blinding

Stage 1: No blinding (open label).

Stage 2: This will be a double-blind trial. If a participant reaches the primary endpoint (development of severe diarrhoea within 3 weeks post HSCT) investigators will be unblinded. If the participant is receiving placebo, they will be given a rescue course of open-label FMT (30–36 capsules taken over 5–6 days, i.e. 6 capsules, 3 BD). This innovative design enables the evaluation of the therapeutic efficacy of FMT (i.e. impact of FMT on diarrhoea) without compromising the validity of the trial’s primary objective.

#### Randomisation

The trial statistician will be responsible for generating the randomisation schedule (stratified for site and conditioning regimen). This will assign a randomisation code to either placebo or the FMT. Participants will be randomised at a ratio of 2:1 (intervention to placebo).

### Study assessments and outcome measures

After consenting to the study, the participants will undergo a baseline assessment, with collection of the following information: UR number, date of birth, sex, height, weight, diagnosis, treatment details, comorbidities, concurrent medications, ECOG performance status.

#### Stage 1 – Safety run in

Safety: Incidence of adverse events (AEs) assessed using the NCI CTCAE v5.0 (when in patient) with focus on treatment emergent adverse events, i.e. those identified to be related to the IP. AEs will be assessed from the time of starting the first course of FMT until 1 month after completion of the second FMT course. These will be assessed daily if the participant is admitted, and weekly when discharged. When an out-patient, assessments will be performed weekly by a member of the study team (phone call to participants). The Safety Monitoring Committee will be responsible for assigning AEs to either the HSCT treatment or IP. If the AE is deemed as probably or definitely related to the IP, they will be defined as a TE-AE.

Adherence/feasibility: Adherence will be defined by the proportion of participants that complete full course of FMT defined by the number of returned (unused) capsules and from participants’ diaries. The IP will be deemed feasible if participants take at least 75% of each course (pre- and post-HSCT).

#### Stage 2 – Efficacy trial

##### Primary outcome

Proportion of participants with severe diarrhoea within 3 weeks of HSCT (severe diarrhoea defined as: 3 consecutive days of Bristol Stool Chart (BSC) 6 + at a frequency of 4 + bowel movements/day above baseline).

##### Secondary outcomes

- Mean duration of BSC 6 + within 3 weeks of HSCT.
- Mean BSC score within 3 weeks of HSCT.
- Mean stool frequency with 3 weeks of HSCT.
- Change in body weight (kg).
- Incidence of FMT treatment-emergent adverse events.
- Proportion of participants that complete full course (as indicator of adherence).
- Incidence of fever (collected from clinical notes).
- Incidence of blood stream infections (defined as positive blood culture, collected from clinical notes).
- Use of supportive care interventions:
  - Empirical antibiotic use (type, dose and duration, collected from clinical notes).
  - Total parenteral nutrition (incidence and duration, collected from clinical notes).
  - Loperamide (incidence, dose and duration, collected from clinical notes).
  - Opioid analgesics (incidence, dose and duration, collected from clinical notes).
  - Other relevant medications used to control symptoms.
- Duration of hospitalisation (days).
- Symptom burden (defined using ESAS-r-CS).

##### Exploratory outcomes

- Gut microbiota composition assessed using microbial genomic sequencing performed on longitudinal stool samples.
- Plasma short chain fatty acid concentrations assessed in longitudinal blood samples.
- Plasma citrulline (biomarker of mucosal barrier injury) assessed in longitudinal blood samples.
- Salivayr metabolome and microbiome.

Safety and adherence will continue to be assessed as per Stage 1.

See Table 3 for study assessment schedule.

**Table 3:** Study assessment schedule

Assessment	Before prophylactic FMT	After prophylactic FMT	Day of HSCT (day 0)	During in-patient stay	Before recovery FMT	After recovery FMT	Day + 21	1 month after
Patient demographics								
Comorbidities								
Medications								
Diagnosis/treatment details								
ECOG performance status								
Adherence								
Bristol stool chart (BSC)			Daily					
Frequency			Daily					
Body weight				2-3x weekly while an in-patient				
Symptom burden			*	*	*	*		*
Adverse events ^								

### Biospecimen collection

Stage 1: Faeces will be collected before and after FMT to confirm engraftment in the safety run in cohort..

Stage 2: Blood, saliva and stool (faeces) will be collected from participants longitudinally throughout the study. A maximum of 2 × 9 ml EDTA blood tubes and 2 × 9 ml SERUM (clotting) blood tubes will be collected at each timepoint. Saliva will be collected using ORAGENE-DNA OG-500 (self collection) tubes twice (sample 1: prior to starting FMT#1, sample 2: after FMT#1, prior to starting conditioning chemotherapy). will be collected using Zymo DNA/RNA shield tubes enabling self-collection by participants and easy transport in the registered mail.

See Table 4a,b for biospecimen collection schedule.

Blood will be collected at the following timepoints (+/- 2 days):

**Table 4a:** Blood collection schedule

Before starting FMT#1	Day of chemotherapy infusion	Day 0 (HSCT)	Post HSCT period	Day of discharge	Day ~ 35*
X	X	X	A maximum of 5x weekly aligning with routine blood draws	X	X

* Aligning with relevant clinic follow up appointment

Stool will be collected at the following time points (+/-2 day depending on the bowel habits of the participant):

**Table 4b:** Stool collection schedule

Before starting FMT#1	Day of chemotherapy infusion	Day 0 (HSCT)	Post HSCT period	Start of FMT #2	End of FMT#2	Day ~ 35*
X	X	X	A maximum of 5x weekly while and in-patient*	X	X	X

* If out-patient, participant will self-collect

### Follow up

At 1 month after the last FMT capsule is taken, participants will complete a final set of assessments to determine symptom burden and to identify any adverse events that may be related to the FMT. ESAS-r-CS will be completed to determine symptom burden. Adverse events will be reviewed and categorised using NCI CTCAE v5.0. Assessments will be performed over the phone by a member of the study team.

### Data management

All data collected from participants in the HSCT-BIOME trial wil be de-identified using their unique Study ID. Data will be collected directly from participants in the form of hard copy Case Report Forms (CRFs) which will be securely stored in a locked filing cabinet that is only accessible to study staff.

Identifiable data (consent forms, pathology reports, etc.) will be de-identified and filed with the study documents. All participant files will be reconciled and stored along with all study materials – both hard copy and electronic – consistent with ICH GCP and applicable regulations regarding the retention and disposal of participant records.

All study data (de-identified) will be scanned into electronic PDFs and stored on a secure, regularly backed-up web-based platform (LabAchives) before being entered into REDCap (Research Electronic Data Capture); a secure web-based application designed to support data capture for research studies [18]. All web-based information transmissions in REDCap are protected via Secure Sockets Layer (SSL) encryption (data entry, survey submission, web browsing, etc.).

### Data analyses and synthesis

All qualitative data will be presented as descriptive data (e.g. participant demographics) and compared between groups using a Chi squared test. Study endpoints will be analysed as follows:

- Effectiveness: Proportion of participants that meet the primary endpoint. This will be compared between the two arms using a Chi squared test.
- Safety: The incidence of all treatment-related AEs and SAEs will be compared between the two groups using a Chi squared test.
- Adherence: The proportion of participants that take all capsules. This will be compared between the two arms using a Chi squared test.
- Exploratory microbiota analyses: Uptake of the FMT will be determined by bacterial genome sequencing performed on faecal samples collected from participants.

### Ethics and dessimination

#### Ethical considerations

This Protocol has been designed to comply with the Declaration of Helsinki and any subsequent amendments, the ICH Guidelines for Good Clinical Practice (CPMP/ICH/153/95) annotated with TGA comments (July 2000), the NHMRC National Statement on Ethical Conduct in Research involving Humans (2007, updated 2018), the policies and procedures of any applicable local guidelines. The trial will be conducted in compliance with the Protocol, International Conference on Harmonisation, Good Clinical Practice (ICH GCP) Guidelines in Australia, and applicable regulatory requirements. Except for an emergency situation in which proper care for the protection, safety and well-being of the study participant requires that an alternative treatment be used, the study shall be conducted exactly as described in the approved protocol.

#### Safety and data monitoring

A Safety Monitoring Committee will be established for the trial, which will include:

- Site PIs.
- Chief investigator.
- Head of Haematology.
- BiomeBank representative(s).

The Safety Monitoring Committee will meet quarterly and review recruitment rates, AE data and other logistical aspects of the trial (e.g. budget). When the Safety Monitoring Committee discussion concerns the treatment of individual participants, the Safety Monitoring Committee consultation process will include the treating clinician of the participant concerned. Participation by the trial statistician and BiomeBank representative will be optional. All discussions about a participant and/or their data, once they are enrolled, will be in a de-identified manner.

If the Safety Monitoring Committee identifies a serious Treatment Emergent Adverse Event (TEAE), they have the discretion to stop the trial or withhold the Investigational Product from the participant. This will be discussed and determined by the Safety Monitoring Committee depending on the severity of the TE-AE and its relationship with the Investigational Product.

#### Dissemination plan

Results will be published in appropriate peer-reviewed journals and presented at national and international scientific meetings (e.g. Haematolgy Society of Australian and New Zealand, Multinational Association for Supportive Care in Cancer, Clinical Oncology Society of Australia, International Human Microbiome Congress, American Society for Clinical Oncology, European Bone Marrow Transplantation Congress, European Society of Medical Oncology, American Society of Haematology). We will also work with community organisations such as Leukaemia Foundation and Myeloma Australia to ensure these data are communicated with consumers.

## Discussion

Fecal microbiota transplantation (FMT) technology is rapidly evolving in its science and form, facilitating its use in vulnerable populations that lack the protective benefits of a healthy microbiome or where traditional methods of administration are complex, or even, contraindicated. FMT is recognised to be beneficial in HSCT recipients, yet its use in the acute phases of HSCT, when its benefits are likely maximised, has been limited. The HSCT-BIOME study will be the first to investigate the efficacy of peri-HSCT delivered oral capsule FMT against the primary outcome measure of severe diarrhoea post HSCT. This study will thus clarify if pre-HSCT delivered FMT can promote microbial resilience against insults experienced during HSCT, and whether post-HSCT delivered capsule FMT can be safely and feasibly administered to immunocompromised patients to improve treatment outcomes.

Whilst our study design remains largely aligned with existing studies, there are unique aspects of the study including the use of peri-HSCT FMT and provision of an encapsulated product. Of particular interest is the decision to include open-label, rescue FMT in situations where participants in the placebo arm reach the primary endpoint. This not only increases participant satisfaction, through accessing the intervention irrespective of randomisation, but also enables both the prophylactic and therapeutic efficacy of FMT to be explored in a single cohort, thus increasing the efficiency and value of the trial.

While the HSCT-BIOME trial primarily focuses on symptoms and clinical outcomes, it will also have a strong mechanistic focus on minimising the depth and duration of GI injury to reduce the sequaelae of associated adverse effects (e.g. infection, malnutrition). Longitudinal collection of blood and stool biospecimens throughout the study will allow for more in depth exploration of the physiological effects induced by the intervention (changes to the microbiome, intestinal health, and reconstitution of immunity), which may be useful in the identification of key attributes related to the host and the FMT that dictate response. These insights will be used to develop next generation, engineered FMT products specifically designed for HSCT.

## Data Availability

No datasets were generated or analysed during the current study.

## Abbreviations

AE: Adverse event
ANC: Absolute neutrophil count
BSC: Bristol stool chart
BSI: Blood stream infection
CALD: Culturally and Linguistically diverse
CDI: Clostridium difficule infection
CRF: Clinical Record Forms
ESAS-r-CS: Revised Edmonton Symptom Assessment System including constipation/sleep
FMT: Faecal microbiota transplantation
GCP: Good clinical practice
GVHD: Graft versus Host Disease
HSCT: Haematopoietic stem cell transplantation
IP: Investigation product
MDB: Multidrug resistant bacteria
NCI CTCAE: National Cancer Institute’s common terminology criteria for adverse events
PI: Principal investigatorRedCAP: Research Electronic Data Capture
SAE: Serious adverse event
SCFA: Short chain fatty acid
SSL: Secure sockets layer
TBI: Total body irradiation
TE-AE: Treatment emergent adverse event
TGA: Therapeutic Goods Administration
